# Berberine modulates AP-1 activity to suppress HPV transcription and downstream signaling to induce growth arrest and apoptosis in cervical cancer cells

**DOI:** 10.1186/1476-4598-10-39

**Published:** 2011-04-15

**Authors:** Sutapa Mahata, Alok C Bharti, Shirish Shukla, Abhishek Tyagi, Syed A Husain, Bhudev C Das

**Affiliations:** 1 Division of Molecular Oncology, Institute of Cytology and Preventive Oncology (Indian Council of Medical Research), I-7, Sector-39, Noida, Gautam Budh Nagar - 201301 India; 2 Department of Biosciences, Faculty of Natural Sciences, Jamia Millia Islamia, New Delhi -110025, India; 3 Dr. B.R Ambedkar Centre for Biomedical Research, University of Delhi (North Campus), Delhi-110007, India

## Abstract

**Background-:**

Specific types of high risk Human papillomaviruses (HR-HPVs) particularly, HPV types 16 and 18 cause cervical cancer and while the two recently developed vaccines against these HPV types are prophylactic in nature, therapeutic options for treatment and management of already existing HPV infection are not available as yet. Because transcription factor, Activator Protein-1 (AP-1) plays a central role in HPV-mediated cervical carcinogenesis, we explored the possibility of its therapeutic targeting by berberine, a natural alkaloid derived from a medicinal plant species, *Berberis *which has been shown to possess anti-inflammatory and anti-cancer properties with no known toxicity; however, the effect of berberine against HPV has not been elucidated.

**Results-:**

We studied the effect of berberine on HPV16-positive cervical cancer cell line, SiHa and HPV18-positive cervical cancer cell line, HeLa using electrophoretic mobility gel shift assays, western and northern blotting which showed that berberine could selectively inhibit constitutively activated AP-1 in a dose- and time-dependent manner and downregulates HPV oncogenes expression. Inhibition of AP-1 was also accompanied by changes in the composition of their DNA-binding complex. Berberine specifically downregulated expression of oncogenic c-Fos which was also absent in the AP-1 binding complex. Treatment with berberine resulted in repression of E6 and E7 levels and concomitant increase in p53 and Rb expression in both cell types. Berberine also suppressed expression of telomerase protein, hTERT, which translated into growth inhibition of cervical cancer cells. Interestingly, a higher concentration of berberine was found to reduce the cell viability through mitochondria-mediated pathway and induce apoptosis by activating caspase-3.

**Conclusion-:**

These results indicate that berberine can effectively target both the host and viral factors responsible for development of cervical cancer through inhibition of AP-1 and blocking viral oncoproteins E6 and E7 expression. Inhibition of AP-1 activity by berberine may be one of the mechanisms responsible for the anti-HPV effect of berberine. We propose that berberine is a potentially promising compound for the treatment of cervical cancer infected with HPV.

## Background

Cervical cancer is the most frequent malignancy in Indian women, and is responsible for substantial morbidity and mortality worldwide [1]. Persistent infection with high-risk human papillomavirus (HR-HPV), most notably types 16 and 18 is an essential prerequisite for the development of cervical cancer [2]. During malignant progression, the HPV integrates into the host cell genome resulting in a loss of expression of the viral E2 gene and over-expression of the two early viral oncogenes E6 and E7, the products of which interfere with the tumor suppressor proteins p53 and Rb respectively. E6 binds and induces ubiquitin mediated degradation of p53 while E7 inactivate pRb leading to dysregulated cell growth [3].

The constitutive expression of HR-HPV E6 and E7 oncogene is mainly dependent on the availability of host cell transcription factors. Activator protein-1(AP-1) which is a heterodimer of a group of structurally and functionally related members of the Jun proteins (c-Jun, JunB, JunD) and Fos proteins (c-Fos, FosB, Fra-1 and Fra-2) found to be constitutively active in cervical cancer. Mutational inactivation of AP-1 consensus sequence within the binding sites of the HR-HPV upstream regulatory region (URR) revealed a complete loss of transcriptional activity of the E6/E7 promoter indicating a key role of AP-1 in HPV-mediated carcinogenesis [4]. Interestingly, AP-1 independently has also been shown to develop carcinogenesis in a variety of tissues [5]. Additionally, studies by our group demonstrated a significant overexpression of constitutively active AP-1 family members in cervical precancer and cancer tissues [6].

The most important risk factor in this cancer is the presence of human papillomavirus (HPV) infection. Conventional therapies like removal of lesions through cryo, laser therapy, excisional surgery, or topical application of formulations of podophyllotoxin, trichloroacetic acid and salicylic acid or 5-fluorouracil, including direct injections of interferon into the lesions may help eliminate the lesions or warts but none of them eradicates the virus. Consequently, recurrence of the lesions, as well as transmission of the virus remains a very significant problem. Since HR-HPV E6 and E7 are the two transforming proteins constantly expressed in transformed cells, they represent ideal targets for development of anti-HPV therapeutics [7]. Antiviral approach against transcriptional inactivation of HPV using herbal derivatives that show minimal or no systemic toxicity could be a promising option to control HPV infection particularly in an early stage of cervical carcinogenesis.

**Figure 1:**
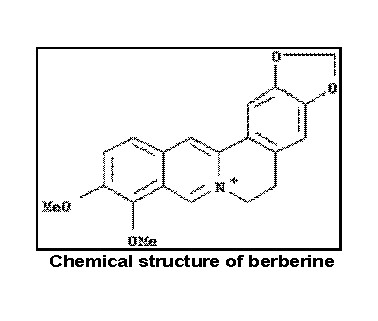
**The chemical structure of berberine**. Source: British booklet on Berberine [8].

Berberine (5, 6-dihydro-9, 10-dimethoxybenzo[g]-1, 3-benzodioxole5,6-aquinolizum) (Figure 1) [8], a natural isoquinoline alkaloid present in roots, rhizome and outer bark of an important medicinal plant species, *Berberis (B. aquifolium, B. vulgaris, B. aristata*, etc.) has been reported to exhibit variety of pharmacological, biochemical and anticancer effects [9]. The medicinal value of berberine is indicated by its use in the Indian Ayurvedic, Unani and Chinese systems of Medicine since time immemorial [10,11]. Berberine has been shown specifically to suppress the growth of a wide variety of tumors including leukemia [12], melanoma [13], epidermoid carcinoma [14], hepatoma [15], oral carcinoma [16] glioblastoma [17], lung [18], prostate [19] and gastric carcinoma [20] and does not have toxic effects on growth and viability of normal cells [14,19,21]. Animal studies have also shown that berberine can suppress chemical-induced carcinogenesis [22], tumor promotion [23] and tumor invasion [18,24]. It also acts as a radiosensitizer of tumor cells but not for normal cells [25]. Though anticancer activity of berberine has been demonstrated [26] but how it mediate these effects is not clearly understood and also, its effect on HPV has not been reported. Therefore, in the present study, we have investigated the effect of berberine on HPV positive cervical cancer cells to examine its anti-viral activity. We show here that both viral transcription and cellular proliferation are strongly affected by berberine which specifically suppresses HPV transcription and constitutively active AP-1 in dose and time dependent manner.

## Materials and methods

### Materials

The HPV16 positive human cervical cancer cell line, SiHa, HPV18 positive human cervical cancer cell line, HeLa and the HPV negative human cervical cancer cell line, C33a were obtained from the American Type Culture Collection (ATCC), USA and were tested and authenticated prior to initiation of experiments and periodically checked for PCR positivity of HPV16 and HPV18 and contaminations to ensure purity of the cell line. DNA hybridization probes, pHPV16 represent unit-length of HPV16 DNA [2] cloned in pBR322, pHPV18 represent unit-length of HPV18 DNA [6] cloned in pBR322, and pHF-A [6] harboring an approximately full-length insert of the fibroblast β-actin gene was kindly provided by L. Kedes (Medical Center, Palo Alto, CA). Custom synthesized and HPLC purified Oligos were procured from M/s Microsynth, (Germany); Polyclonal antibodies to AP-1, hTERT, Caspase-3, Rb, PARP-1 and Monoclonal antibodies to HPV16E6/18E6, HPV16E7, HPV18E7, p53 were purchased from Santa Cruz Biotechnology (Santa Cruz, CA). DMEM, FCS, MTT, and Penicillin-Streptomycin solution were obtained from Sigma (St Louis, MO). All other reagents were of analytical molecular biology grades.

### Cell culture

Cells were maintained in Dulbecco's modified Eagle's medium (DMEM), supplemented with 10% heat- inactivated fetal calf serum and 1% penicillin/streptomycin in CO_2 _incubator with a humidified atmosphere of 95% air and 5% CO_2 _at 37°C.

### Berberine

Commercially available berberine (Sigma) was freshly dissolved in DMSO [maximum concentration, 0.5% (v/v)], which was then added to complete cell culture medium prior to addition to subconfluent cells. Cells treated with vehicle only (DMSO, 0.5% in media) served as control.

### Lymphocytes isolation

Peripheral blood lymphocytes were isolated from heparinized blood collected from healthy volunteers by standard method of Ficoll-Hypaque gradient centrifugation as described by Bharti et.al [27]. These cells were used for subsequent MTT assay.

### MTT assay

The cytotoxic effects of berberine against SiHa, HeLa, C33a and Lymphocytes were determined by MTT dye uptake method. The cells were incubated in triplicate in a 96-well plate in the presence or absence of indicated test samples in a final volume of 0.1 ml for 24 h, 48 h and 72 h at 37°C in a CO_2 _incubator. Thereafter 0.025 ml of MTT solution (5 mg/ml in PBS) was added to each well. After 2 h incubation at 37°C, lysis buffer (20%SDS 50% Dimethyl Formamide) was added, and the extract was incubated overnight at 37°C for solublization of formazan crystals. The OD at 570 nm was measured using a 96-well multiscanner autoreader (Biotek, Winooski,Vermont) with the lysis buffer serving as blank. The percentage of cell viability was calculated using the following formula: Percentage cell viability = (OD of the experiment samples/OD of the control) × 100.

### RNA Extraction and Northern blotting

The cellular RNA were extracted following treatment of SiHa and HeLa cells with 0, 50, 100 and 250 μg/ml berberine for 24 h by using TRI Reagent according to the manufacturer's instruction. The quality of RNA was estimated by electrophoresis using 2 μl of RNA solution on an ethidium bromide-stained 1% agarose gel in 3-[N-morpholino] propane-sulfonic acid (MOPS) buffer. Concentration of RNA was estimated by Nanodrop (NanoDrop Tech, USA). The probes were labeled by the random-priming method using random primer labelling kit (Genei, Bangalore, India) and northern blotting was carried out using standard protocols [28]. Briefly, approximately 15 μg of RNA was resolved on 1% agarose- MOPS formaldehyde gel. Capillary blotted Nylon membrane (IMMOBILON-NY+, Millipore, Bedford, MA) was then UV crosslinked (Hoefer UVC 500 ultraviolet crosslinker, Amersham Biosciences) and washed in 6X SSC, air dried, and finally exposed in phosphorimager (Fujifilm FLA-5100) after pre-hybridization and hybridization in Perfect HYB-PLUS (Sigma Inc, USA) solution as suggested by manufacturer's protocol.

### Electrophoretic mobility shift assay

For electrophoretic mobility shift assay (EMSA), the following oligonucleotides were used: AP-1 consensus sequence 5'-CGCTTGATGACTCAGCCGGAA-3' [29], Oct-1 consensus oligonucleotide 5'-TGTCGAATGCAAATCACTAGAA-3'[30] and Sp-1 consensus sequence 5'-ATTCGATCGGGGCGGGGCGAG-3'[31]. Cells treated with different concentration of berberine for different time intervals were harvested and then nuclear extracts were prepared as described earlier [6]. The protein concentration of the extracts was measured by the spectrophotometric method using Nanodrop spectrophotometer ND-100. EMSA was performed using 10 μg of nuclear extract as described previously [6]. For supershift assays, 2 μg of polyclonal antibodies (Abs) directed against the Jun/Fos members (Santa Cruz Biotechnology Inc., Santa Cruz, CA) were added and the reaction mixture was further incubated for 45 mins at 4°C. The following antibodies were used: c-Jun (epitope corresponding to amino-terminal domain of mouse c-Jun p39); JunB (epitope corresponding to carboxy terminal domain of mouse JunB); JunD (epitope corresponding to carboxy terminus of mouse JunD); c-fos (epitope corresponding to a highly conserved domain of c-fos p62 of human origin); FosB (epitope corresponding to amino acids within the central domain of the FosB protein of mouse origin); Fra-1(epitope corresponding to amino terminus of Fra-1 of rat origin) and Fra-2 (epitope corresponding to carboxy terminus of Fra-2 of human origin). The DNA-protein complexes were then resolved on 4.5% nondenaturing polyacrylamide gel, dried and either exposed overnight to Kodak X-Omat Films (Kodak India Ltd., India) or visualized by PhosphorImager (Fujifilm FLA-5100) using Multi Gauge-ver 3.x anlaysis software. The quantitative densitometric analysis was performed using Alpha Ease FC version 4.1.0 (Alpha Innotech Corporation, IL).

### Western blotting

Whole cell lysate (50 μg/lane) were resolved by SDS-PAGE, electrotransferred to Immobilon-P membranes (Millipore Corporation, Bedford, MA). The membrane was blocked with 10% non-fat milk and incubated overnight in PBS with 5% milk, 0.05% Tween-20 and probed with polyclonal rabbit primary antibodies of the corresponding family members (see Electrophoretic mobility shift assay for AP-1) at 4°C. These blots were washed, incubated with HRP- anti-rabbit IgG secondary antibodies and visualized by Luminol detection kit (Santa Cruz Biotech, USA). Membrane was re-probed for β-actin expression as an internal control. The ratio of the specific proteins to β-actin was calculated.

### Flow cytometric analysis of apoptotic cell death by Annexin V-FITC

Cells were treated with berberine for 24 h. The cells were then harvested, washed with PBS and incubated with AnnexinV-conjugated fluorescein isothiocynate (FITC) and propidium iodide (PI) for cellular staining as described in AnnexinV-FITC apoptosis detection kit (BD Biosciences) manufacturer's instructions. The stained cells were then analyzed by FACS. The number of 10000 events was acquired and the cells were properly gated for analysis using FACSAria instrument equipped with Flowjo software (Becton-Dickinson Biosciences, San Jose, CA).

### Quantitation of Caspase- 3 Activity

The activity of caspase-3 was measured using the active caspase-3 apoptosis kit (BD Pharmingen, USA) following the manufacturer's protocol. Briefly, cells were treated with different doses of berberine for 24 h or for different time intervals and were harvested by pooling attached and detached cells were pelleted with centrifugation at 200 × *g *for 5 min at 4°C. The cells were permeabilized, fixed, and stained for active caspase-3 (PE-conjugated) as described in manufacturer's protocol (BD Biosciences).

### Measurement of mitochondrial membrane potential

Cells were plated onto a 60-mm tissue culture plate at subconfluent density. After 24 h incubation cells were exposed to different doses of berberine and incubated with 5 μM JC-1 fluorescence dye for 30 min in CO_2 _incubator and washed several times with PBS pre-warmed at 37°C. Mitochondrial membrane potential was evaluated qualitatively under a fluorescence microscope (Olympus IX81) using 568 nm filter.

### Statistical analysis

All experiments were conducted in triplicate for at least three times. The statistical significance of difference between control and treated groups was analyzed by the one- way ANOVA (Holm-Sidak method) (Sigma Stat 3.5, Systat software Inc., CA). The difference was considered significant when the *p *value was less than 0.05.

## Results

### Berberine selectively downregulates constitutively active AP-1 in HPV16 positive cervical cancer cells, SiHa

**Figure 2:**
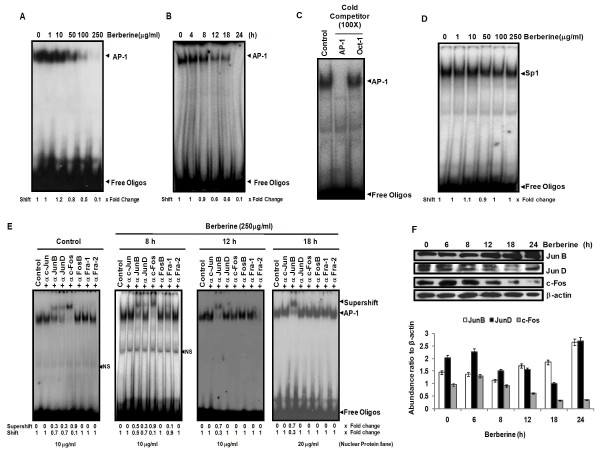
**Berberine specifically inhibits AP-1 DNA binding activity in HPV-16 positive cervical cancer cells**. SiHa cells treated with indicated concentrations of berberine for 24 h ***(A)***, or treated with 250 μg/ml berberine for indicated durations ***(B) ***were assayed for AP-1 DNA binding activity by EMSA and fold change in DNA binding was calculated by densitometric evaluation of shifted band. ***C***, *Binding of AP-1 to its consensus oligo probe is specific*. Untreated SiHa nuclear proteins and labeled AP-1 probe were incubated in the presence of 100 fold molar excess of unlabeled homologous AP-1 probe or heterologous Oct-1 probe and assayed for AP-1 specific binding by EMSA. ***D***, *Berberine does not alter basal activity of other ubiquitous transcription factor Sp1*. Nuclear proteins (10 μg) of berberine- treated cells were checked for Sp1 DNA binding activity by EMSA. ***E***, *Effect of Berberine on composition of AP-1 in DNA-binding complex*. Nuclear proteins of berberine (250 μg/ml) treated cells for different time periods were checked for various AP-1 proteins present in functional AP-1 complex by supershift assay (*Excess of nuclear proteins of 18 h treated cells were used to reveal participation of AP-1 members.) ***F***, *Effect of berberine on the expression of AP-1 family proteins*. Cellular proteins isolated from berberine (250 μg/ml) treated SiHa cells for indicated time durations were examined for the expression of AP-1 family proteins members. β-actin was used as loading control. The abundance ratio to β-actin was analyzed by densitometry. The data are expressed as the mean ± SD of 3 independent experiments.

To assess anti-HPV activity of berberine, we investigated the effect of berberine on AP-1, which is constitutively active in cervical cancer and plays an indispensable role in transcriptional regulation of HPV oncogenes. HPV16 positive cervical carcinoma cells, SiHa were treated with different concentrations of berberine for 24 h and the nuclear protein (10 μg) extracted were examined for AP-1 DNA-binding activity by EMSA. Results revealed a dose-dependent decrease of AP-1 DNA binding activity in berberine-treated cells (Figure 2A). Inhibition was apparent at 50 μg/ml and a maximum inhibition was obtained at 250 μg/ml. Densitometric analysis of the retarded bands showed a 10 fold decrease in AP-1 DNA-binding activity. Further analysis of time kinetics of berberine-induced AP-1 inhibition for different time periods revealed a reduced AP-1 DNA binding activity by 12 h which declined further and disappeared by 24 h (Figure 2B). Specificity of AP-1 DNA binding was confirmed by cold competition assay using 100 fold molar excess of a homologous (AP-1) probe which resulted in disappearance of retarded complex whereas it remained unaffected by addition of heterologous (Oct-1) probe (Figure 2C). Similarly, effect of berberine on general transcription was checked by examining nuclear protein (10 μg) of berberine-treated cells for binding to Sp1, a transcription factor that is ubiquitously active in majority of cells (Figure 2D). Results showed no inhibitory effect of berberine on Sp1 DNA binding activity. The results, thus establish that berberine selectively suppresses constitutively active AP-1 in a dose and time-dependent manner in cervical cancer cells.

### Berberine alters the heterodimerization pattern of AP-1 and differentially modulates expression of Jun and Fos family proteins

To determine the effect of berberine on composition of AP-1 complex and to dissect out the AP-1 protein most sensitive to berberine treatment, we performed supershift assays on SiHa nuclear proteins treated with berberine (250 μg/ml) for 8, 12 and 18 h. Under normal conditions, AP-1 consists of c-Fos, JunB, and JunD (Figure 2E) in its active DNA-binding complex and over 60% of the supershifted band was formed by c-Fos only, while other two members, JunB, and JunD, contributed moderately, but other Fos family members (FosB, Fra-1, Fra-2) as well as c-Jun did not participate in active AP-1 complex. Interestingly, nuclear protein extracted from berberine treated SiHa cells after 12 h displayed exclusive involvement of JunB in the binding activity (~70%) with no involvement of either JunD/c-Jun or any of c-Fos family members in active AP-1 complex (Figure 2E). These results suggest that berberine-induced AP-1 inhibition is primarily mediated through exclusion of c-Fos family of proteins and JunD from active AP-1 complex whereas JunB DNA appears to form homodimer.

In order to assess the possibility of decreased involvement of c-Fos and JunD in active AP-1 complex could be due to their reduced expression, we analyzed expression of c-Fos, JunB, and JunD by western blotting at different time intervals following berberine treatment. As shown in Figure 2F, berberine-treated cells demonstrated reduced expression of c-Fos but interestingly though JunD also showed reduced expression by 12 h of treatment, its expression got recovered by 24 h. On the other hand, a compensatory and marginal increase in the expression of JunB was observed in berberine-treated cells. These results, therefore, indicate berberine-induced inhibition of AP-1 is primarily mediated through inhibition of c-Fos expression and its exclusion from active complex in cervical cancer cells.

### Berberine inhibits AP-1 activity and reduces the expression of c-Jun and c-Fos in HPV18 positive cervical cancer cells

**Figure 3:**
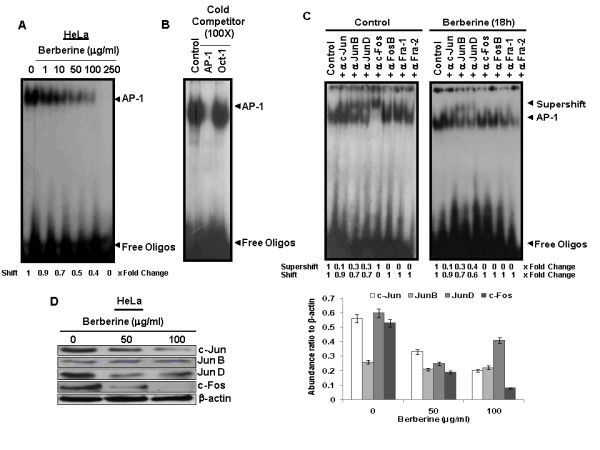
**Berberine specifically downregulates constitutive active AP-1 in HPV18 positive cervical cancer cells**. ***A***, *Dose-dependent downregulation of AP-1 binding activity*. Nuclear proteins of HeLa cells treated without or with indicated concentrations of berberine for 24 h were prepared and assayed for AP-1 DNA binding activity by EMSA. As indicated in "Methods" fold change was calculated following densitometric evaluation of shifted band. ***B***, *Binding of AP-1 to its consensus oligo probe is specific*. Binding specificity of AP-1 to its consensus probe was examined by cold competition in a binding reaction of untreated HeLa nuclear proteins and labeled AP-1 probe adding 100 fold molar excess of unlabeled homologous AP-1 probe or heterologous Oct-1 probe. ***C***, *Berberine changes composition of AP-1 DNA-binding complex in HeLa cells*. Equal amount of nuclear extracts (10 μg/lane) isolated from untreated as well as treated (100 μg/ml berberine for 18 h) HeLa cells were checked for binding partners in functional AP-1 complex by co-incubating these extracts individually with different antibodies as described in 'Methods'. (*Excess of nuclear proteins used to reveal participation of AP-1 members.) ***D***, *Effect of berberine on the expression of AP-1 family proteins*. HeLa cells treated with berberine for indicated doses and protein isolated from these cells were subjected to western blot analysis as described in "Methods". β-actin was used as loading control. The abundance ratio to β-actin was analyzed by densitometry. The data are expressed as the mean ± SD of 3 independent experiments.

We further looked into the effect of berberine on AP-1 activity in cervical cancer cells, HeLa that harbor HR-HPV18 infection and also express constitutively active AP-1. Nuclear proteins of HeLa cells which were treated with varying concentrations of berberine demonstrated a similar specific dose-dependent inhibition of AP-1 DNA binding by EMSA (Figure 3A, B). HeLa cell nuclear proteins were also examined for the composition of activated AP-1 complex by supershift assays revealed presence of c-Jun, JunB, JunD and c-Fos (Figure 3C) in active AP-1 complex whereas FosB, Fra-1,and Fra-2 showed no participation in the AP-1 complex formation. As compared to SiHa cells, where there is no involvement of c-Jun, we have found presence of c-Jun in HeLa cells but to a lesser extent. To determine the effect of berberine on specific AP-1 proteins involved in active complex, nuclear protein of HeLa cells treated with berberine were examined by supershift assays which displayed exclusive involvement of JunB and JunD in the binding activity with no involvement of c-Fos (Figure 3C). These results suggest that c-Fos is the most sensitive AP-1 member and its exclusion from active AP-1 complex contributes maximally to the loss of AP-1 activity in berberine-treated cells. Further investigation of AP-1 protein expression revealed a dose-dependent loss of c-Fos and c-Jun in berberine treated cell (Figure 3D). These observations collectively indicated exclusion of Fos member from active complex and loss of AP-1 activity are primarily mediated through loss of expression of c-Fos and c-Jun in berberine-treated cells.

### Berberine downregulates HPV16 and HPV18 transcription, suppressed E6, E7 and hTERT expression and increased p53 and Rb expression in cervical cancer cells

**Figure 4:**
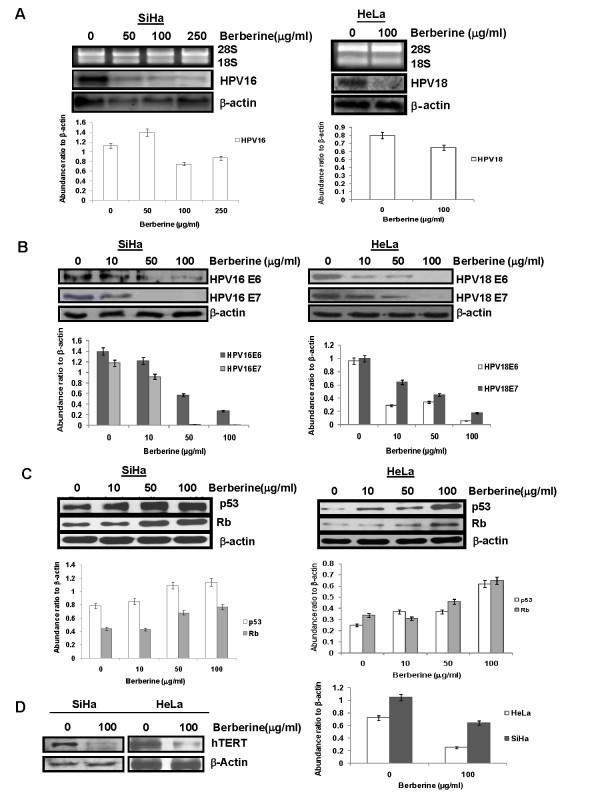
**Berberine downregulates HPV oncogene expression, increased p53, Rb levels and inhibit hTERT expression in cervical cancer cells**. ***A***, *Effect of berberine on HPV16 and HPV18 mRNA expression*. Northern blots (middle panels) of HPV16 positive SiHa cells and HPV18 positive HeLa cells incubated with indicated concentrations of berberine. Quantity and quality of total RNA (15 μg/lane) extracted was examined on agarose gel (upper panels). The membrane was rehybridized with β-actin-specific DNA probe as internal control to assess equal loading (lower panels). ***B***, *Effect of berberine on the expression of HPV E6 and E7 oncoproteins*. Representative immunoblots of HPV16E6 &E7 and HPV18E6 &E7 in SiHa and HeLa cells respectively treated with berberine for indicated doses. β-actin was used as loading control. ***C***, *Effect of berberine on the expression of p53 and Rb proteins*. HPV16 positive SiHa cells and HPV18-positive HeLa cells were treated with different concentration of berberine. The cells were harvested after 24 h to obtain whole cell extracts, which were analyzed for p53 and Rb expression by Western blotting. Equal sample loading was confirmed by determining the β-actin expression levels. ***D***, *Effect of berberine on the expression of hTERT*. Immunoblots of cellular proteins isolated from SiHa and HeLa cells treated with berberine. β-actin was used as loading control. The abundance ratios to β-actin were analyzed by densitometry. The data are expressed as the mean ± SD of 3 independent experiments.

To investigate, whether inhibition of AP-1 by berberine has any impact on the viral transcription, total RNA was extracted from the SiHa and HeLa cells following treatment with different concentrations of berberine for 24 h and northern blotting was performed using HPV16-DNA and HPV18-DNA probes respectively. The results revealed a concentration dependent decline in HPV16-specific transcripts in berberine-treated SiHa cells (Figure 4A). Berberine at 50 μg/ml was found to significantly downregulate viral transcription and strongest reduction was detected in cells treated with 250 μg/ml. A decline in HPV18-specific transcripts was also observed in berberine-treated HeLa cells (Figure 4A). Suppression of HPV transcription was found to be selective since expression of house keeping gene, β-actin was not affected in both the cells.

We then proceeded to investigate the expression level of HPV oncogenes, E6 and E7 after berberine treatment. Data from western blotting analysis showed that the expression of HPV16E6, HPV16E7, HPV18E6 and HPV18E7 were significantly suppressed by berberine in cervical cancer cells in a dose-dependent manner (Figure 4B).

The two most essential cell cycle regulators and tumor suppressor proteins, p53 and Rb being the targets of high-risk HPV E6 and E7 oncoproteins respectively, we also examined the status of p53 and Rb expression in SiHa and HeLa cells. Both of these cervical cancer cells expressed p53 and Rb at low levels which showed a dose- dependent increase in expression following treatment with berberine (Figure 4C).

Since two viral oncoproteins, E6 and E7 encoded by HR-HPVs contribute to immortalization of primary human epithelial cells through the induction of telomerase activity by stimulating transcription of the catalytic subunit of telomerase, hTERT, we examined whether suppression of HPV transcription and reduced expression of viral oncogenes due to berberine also result in altered expression of hTERT. Cellular proteins (50 μg) extracted from SiHa and HeLa cells were incubated in the absence or presence of berberine (100 μg/ml for 24 h) was checked for hTERT expression using western blotting. As depicted in Figure 4D, high expression of hTERT protein was observed in untreated cells which decreased significantly upon berberine treatment in both the cells.

### Berberine decreases cell viability and induce growth inhibition in cervical cancer cells

**Figure 5:**
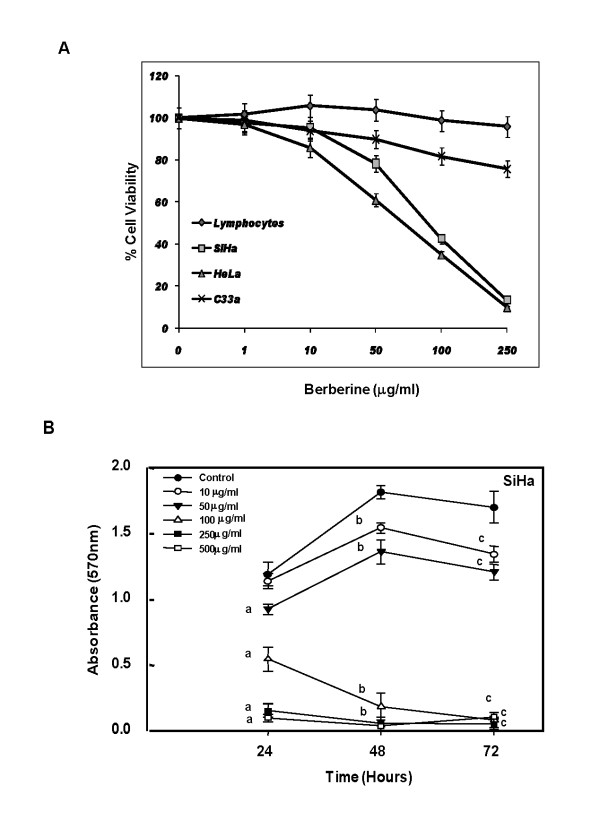
**Berberine treatment results in dose-dependent loss of cell viability and induces growth inhibition in cervical cancer cells**. ***A***, *Percent cell viability of cells treated with berberine for 24 h*. Cervical cancer cells and normal peripheral blood lymphocytes were treated with indicated doses of berberine in triplicates and the viability was measured at different time intervals by MTT assay as described in "Methods". ***B***, *Growth curve of berberine-treated SiHa cells at different time points reflecting cytotoxic and cytostatic effects at different concentration*. Error bars indicates SD. ^a^*p*- < 0.001 is compared to untreated control at 24 h, ^b^*p*- < 0.001 is compared to untreated control at 48 h, ^c^*p*- < 0.001 is compared to untreated control at 72 h.

Activation of AP-1 along with increased expression of viral oncoproteins and telomerase are all critical prerequisites for growth promoting and cell survival mechanisms of cervical cancer cells. Therefore, we were interested to check how does inhibition of these factors translates onto cell survival and growth of berberine-treated cells. For this, cells were treated with different concentration of berberine for 24 h and their viability was checked by MTT assay. As indicated in Figure 5A, Treatment of berberine with varying concentration resulted in concentration-dependent loss of cell viability of both SiHa and HeLa cells with 50% inhibitory dose (ID_50_) of approximately 90 μg/ml for SiHa and 75 μg/ml for HeLa cells and maximal effect was observed at 250 μg/ml. SiHa cells were also checked for their growth kinetics at 24, 48 and 72 h in the absence or presence of different concentration of berberine. As summarized in SiHa cell growth curves in the presence of berberine (Figure 5B), berberine at as low as 10 μg/ml could retard the growth of cervical cancer cells. Berberine at concentration higher than 50 μg/ml resulted in reduced cell viability drastically and cultures did not recover within 72 h. Though berberine inhibits cell proliferation of HPV positive cervical cancer cells, however, in case of HPV negative cervical cancer C33a cells we did not find significant inhibitory effect of berberine on cell viability (1-24% inhibition) (Figure 5A). Treatment of lymphocytes with berberine also results in a non significant inhibitory effect on cell viability (1-4%) at the higher concentrations of berberine (100 μg/ml and 250 μg/ml) after 24 h of treatment (Figure 5A). These data indicates that berberine has a better cytotoxic effect on HPV positive human cervical cancer cells.

### Berberine-induced growth inhibition is mediated through induction of apoptosis

**Figure 6:**
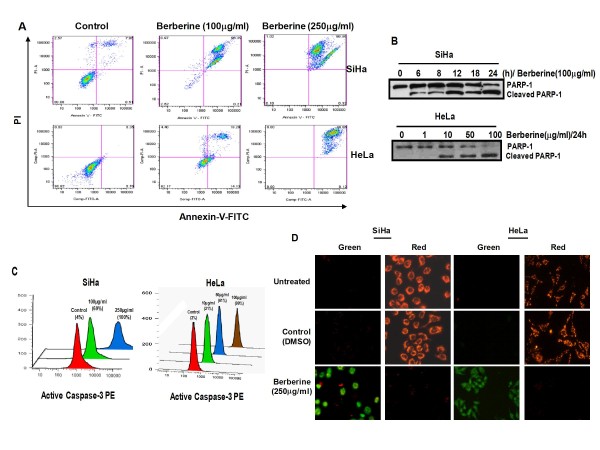
**Berberine induces apoptosis, activation of Caspase 3 and loss of mitochondrial membrane potential in cervical cancer cells**. ***A***, *Flowcytometric analysis of SiHa and HeLa cells treated with berberine for 24 h*. Treated cells were examined for apoptotic cells using Annexin V-FITC apoptosis detection kit. ***B***, Immunoblot analysis of cellular protein isolated from SiHa and HeLa cells treated with berberine for PARP-1 expression as described in "Methods". ***C***, *Flowcytometric analysis of SiHa and HeLa cells for active caspase-3*. Percentage in histograms shows proportion of cell with active caspase-3 after berberine treatment. ***D***, *Immunofluorescence photomicrograph of JC-1 stained untreated, control (DMSO only) and berberine treated SiHa and HeLa cells*. Cells appearing in red channel indicate intact mitochondrial transmembrane potential, whereas cells appearing in green channel indicate loss of mitochondrial membrane potential.

To understand the mechanism of berberine-induced growth inhibition and to examine whether berberine-induced inhibition of cervical cancer cells was associated with the induction of apoptosis, SiHa and HeLa cells were treated with berberine and berberine-induced apoptosis was assessed using Annexin V-PI staining of the treated cells that identify specifically the cells undergoing apoptotic cell death and start expressing phosphatidylserine on their cell surface. As shown in Figure 6A, cells treated with berberine had a very high Annexin V staining and were also positive for PI, a phenotype generally expressed by early apoptotic cells when compared to untreated cells.

To further dissect the berberine-induced apoptotic mechanism, we checked the effect of berberine on Poly (ADP-ribose) polymerase (PARP-1) cleavage, the downstream substrate of active caspase 3. Berberine-treated whole cell lysates (50 μg) of SiHa and HeLa cells were probed for the analysis of PARP-1 by western blotting which showed cleavage of 116 kDa intact PARP-1 into 85 kDa fragment in both the cells (Figure 6B). The quantitation of cells for active caspase 3 by flow cytometry revealed 70% cells positive by 24 h when treated with 100 μg/ml berberine and almost all cells had active caspase-3 when treated with 250 μg/ml of berberine in SiHa cells (Figure 6C). About 99% cells were positive for active caspase-3 in HeLa cells treated with 100 μg/ml berberine for 24 h (Figure 6C).

Since loss of mitochondrial membrane potential is the primary target for majority of extrinsic apoptotic signals, we checked the integrity of mitochondrial membrane using metachromatic dye, 5,5'6,6' tetrachloro-1,1'3,3' tetraethylbenzimidazolylc iodide (JC-1), which stains the mitochondria red when their membranes are intact (polarized) whereas they give green fluorescence with depolarized membranes. Figure 6D clearly demonstrates that in cells treated with a high concentration of berberine (Figure 6D, *bottom panels*) mitochondria lost their membrane potential and thus proceeded through apoptosis.

## Discussion

Host cell derived transcription factor, AP-1 binds to long control region or upstream regulatory region (URR) of HPV, plays an essential role in HPV-mediated host cell immortalization and oncogenic transformation. Site- directed mutagenesis of AP-1 binding sites within the URR regions [32] and stable infection assays in raft culture [33] have established an indispensible role of AP-1 in initiating and maintaining the expression of two essential high risk HPV oncoproteins E6 and E7 during cervical carcinogenesis. Earlier studies from our group and others have demonstrated overexpression and constitutive activation of AP-1 in cervical cancer cells and the DNA binding affinity of AP-1, as well as the expression of its constituent members, varies as a function of the severity of cervical lesions [6,34]. Thus transcription factor, AP-1 can be considered as potential therapeutic targets for cervical cancer. In the present investigation, we show that a naturally occurring isoquinoline alkaloid, berberine, selectively suppress expression of AP-1 transcription factor in a dose and time dependent manner. Inhibition of AP-1 was accompanied by suppression of HPV transcription and oncogene expression as well as inhibition of downstream telomerase component, hTERT. Berberine-mediated inhibition of growth promoting signals culminated in growth inhibition and loss of cell viability through induction of apoptosis in cervical cancer cells. Our results demonstrated a dose-dependent selective suppression of AP-1 activity by berberine which was accompanied by suppression of c-Fos and JunD expression and their reduced involvement in functional AP-1 complex in HPV positive cervical cancer cells irrespective of infecting HR-HPV types whereas JunB that also participated in an active AP-1 complex remained unaffected. Comparison between the two cell lines revealed a specific effect of berberine on c-Fos and c-Jun resulting in their exclusion from the functional AP-1 complex which could be partly due to downregulation of their respective expression levels. Earlier studies also described berberine-induced inhibition of AP-1 in murine tumor models as well as hepatic, breast and oral cancer cells [16,24,35], but the mechanism of its inhibition remained unclear. Recent study shows that inhibitory effect of berberine could be mediated through inhibition of c-Jun that suppresses expression of downstream gene, cyclin D1 and results in cell cycle arrest [36]. However, in HPV16 positive SiHa cells or HPV18 positive HeLa cells it appears that berberine is not executing its effect through this mechanism as involvement of c-Jun in active AP-1 is negligible [6]. On the contrary, expression of c-Fos which is the major partner of active AP-1 dimer was the target of berberine and was found to be the most sensitive among all AP-1 proteins. Although further experiments using selective inhibition of c-fos and JunD by specific siRNA or reporter assays comparing the different homodimers and heterodimers of Jun and Fos family members are required to validate the significance of altered AP-1 composition, the present observations do support berberine as a preferred anti-HPV therapeutic molecule for cervical carcinogenesis. Rapidly growing amount of data from experimental, clinical and animal studies reveal that c-Fos appears to have strong oncogenic activity and is frequently overexpressed in almost all tumor cells [5]. Our earlier study demonstrated c-Fos as a major AP-1 member which showed high expression in cervical carcinogenesis [6]. In an ingenious experiment where c-Fos was ectopically over expressed through stable transfection of nontumorigenic HeLa-fibroblast hybrid 444 cells, it induced tumorigenity. This reiterates the tumorigenic role of c-Fos [37]. AP-1 has been shown to be an important target for anti-oxidant mediated action on cervical cancer cells [28]. However, the mechanism of their action may differ as antioxidants like PDTC enhances AP-1 binding and elicits up-regulation of c-Fos and c-Jun expression. Instead of acting directly on c-Fos it results in upregulation of Fra-1 which has antagonistic role to c-Fos and prevents its involvement in formation of functional AP-1 complex [28]. Though the mechanism(s) underlying berberine-induced inhibition of c-Fos expression is unclear, studies on vascular smooth muscle cells demonstrated that berberine can inhibit c-Fos expression by inhibiting ERK1/2 [38,39], the upstream kinases responsible for c-Fos expression through transcription factor TCF/Elk-1 [40].

The gradual but distinct increase in JunB protein expression after berberine treatment strongly support the tumor suppressor activity of JunB as it was earlier reported that JunB and JunD can negatively regulate cell proliferation [41] and has an opposite effect on gene expression. Furthermore, JunB is also known to be a weaker transactivator than c-Jun [42]. Though JunB is essential for the HPV18 P105 promoter activation [32], there may be possibility that interaction with other proteins may inhibit DNA binding due to direct protein-protein interaction [43] thus, negative interference between proteins, either c-Jun or JunD may be one of the reasons of decrease in AP-1 DNA binding activity.

Our results indicate that berberine can effectively suppress HPV transcription and thus could inhibit the expression of its two oncogenes, E6 and E7 that are critically involved in cellular transformation. Spatial and temporal expression of these viral genes is tightly controlled by specific cognate sequences in URR that bind specific transcription factors of the host cells. The sequence analysis of viral URR region which controls the expression of these oncogenes demonstrates presence of multiple AP-1 binding sites [44] and thus indicates a direct involvement of this transcription factor in oncogenic transformation. Suppression of HPV transcription by berberine, therefore, could be the direct outcome of inhibited AP-1 activation in cervical cancer cells. Apart from targeting p53 and pRB, E6 and E7 have been demonstrated to induce transcription of hTERT, the active component of telomerase responsible for its catalytic activity [45]. Berberine-induced inhibition of viral transcription was associated with suppressed hTERT expression; hence berberine could also target telomerase activity in cervical cancer cells, which we have shown earlier to be an important marker for cervical carcinogenesis [46]. Earlier study on human leukemia cells also provided the evidence that berberine could inhibit telomerase by directly inhibiting expression of its components nucleophosmin/B23 [47] and thus could effectively suppress overall activity independent of HPV involvement. Collectively these observations indicate that berberine could effectively target survival advantage rendered by telomerase expression in HPV-infected cervical cancer cells and could suppress cell proliferation.

In addition to its inhibitory effects on HPV transcription, berberine also antagonizes cell proliferation. Our results demonstrate two distinct concentration-dependent growth inhibitory effects of berberine on cervical cancer cells. Berberine at 50 μg/ml or lower suppressed proliferation whereas at concentration higher than 50 μg/ml resulted in dose-dependent apoptosis. Similar concentration-dependent biphasic effects have been reported earlier [48]. Similar to the cytotoxic/cytostatic effect of berberine observed in present investigation especially in cancer cell lines in contrast to normal lymphocytes, a comparative analysis of studies performed on various human cancer cell lines and primary cultures using purified berberine revealed a differential sensitivity of various cancer cell types whereas normal cells remained unaffected (Table 1). Interestingly, majority of studies performed on cervical cancer cells showed requirement of high concentration of berberine for manifestation of its cytotoxic effect [26,49,50] which could be ascribed to viral etiology of cervical cancer and overexpression of viral oncoproteins E6 and E7 that may effectively override cellular checkpoints. However, it was also observed that the effective cytotoxic doses were always less in HPV positive cells as compared to HPV negative cervical cancer cell, C33a that have undergone cellular transformation independent of viral infection. The reason for such a dichotomy in berberine's effect in cervical cancer cells is unclear.

**Table 1:** Comparative analysis of *in vitro *studies performed for assessing anti-cancer properties of purified berberine (>99%) in various human cancer and normal cells

Cells Type (Human)	Cell Line	Concentration Range Tested		Percent Cell Death at max. Dose after 24 h (Approx; %)	**IC**_**50 **_**(μg/ml) after 24 h**	References
					
		μM	μg/ml			
**Tongue squamous carcinoma**	**SCC-4**	15 - 100	5.58 - 37	45	28 (48 h)	[55]
**Prostrate carcinoma**	**LNCaP**	10 - 100	3.7 - 37	35	>37	[19]
		5 - 100	1.8 - 37	65	22.3	[56]
	**DU145**	10 - 100	3.7 - 37	40	>37	[19]
	**PC-3**	10- 100	3.7 - 37	70	18.6	[19]
		5 - 100	1.8 - 37	50	37	[56]
**Oral squamous cell carcinoma**	**HSC-3**	5 - 75	1.8 - 28	82	6.7	[57]
**Non-small cell lung carcinoma**	**A549**	25 - 100	9.3 - 37	55	28	[21]
	**H1299**	25 - 100	9.3 - 37	50 (48 h)	37 (48 h)	[21]
**Nasopharyngeal carcinoma**	**5-8F**	2.5 - 100	0.93 - 37	50	37	[58]
**Leukemia**	**HL-60**	5 - 60	1.8 -22	70	<11	[59]
**Hepatoma**	**KIM-1**	0.01 - 100	0.0037 - 37	35	14.88	[35]
	**Hep3B**	0.32- 320	0.119 - 119	8.5	Not defined	[60]
	**HA22T/VGH**	0.32 - 320	0.119 - 119	1	Not defined	[60]
	**HepG2**	28 - 107	10 - 40	95	13 (48 h)	[61]
		1 - 10	0.37 - 3.7	70	0.52	[62]
		0.32 - 320	0.119 - 119	6	ND	[60]
**Glioma**	**U87**	1 -20	1 - 7.4	40	>7.4	[63]
	**T98G**	134 - 538	50 - 200	60	134	[17]
**Gastric carcinoma**	**SNU-5**	50 - 200	18.6 - 74	95	17.85	[20]
**Embryonic kidney**	**HEK-293T**	1 - 10	0.37 - 3.7	35	5	[62]
**Epidermoid carcinoma**	**A431**	5 - 75	1.8 - 28	45	>28	[14]
**Breast carcinoma**	**MDA-MB-231**	1 - 10	1 - 3.7	0	Not defined	[24]
**Cervical carcinoma**	**C33a (HPV-)**	**2.7 - 672**	**1 - 250**	**24**	**>250**	**Current study**
	**CaSki (HPV16+)**	50 - 150	18.6 - 55.8	80	42	[26]
	**SiHa (HPV16+)**	**2.7 - 672**	**1 - 250**	**87**	**90**	**Current study**
	**HeLa (HPV18+)**	0.27 - 403	0.1 - 150	ND	6	[49]
		0.1 - 10	0.037 - 3.7	No effect	Not defined	[64]
		33.5 - 269	12.5 - 100	50	100	[50]
		1 - 10	0.37 - 3.7	48	4	[62]
		**2.7 - 672**	**1 - 250**	**90**	**75**	**Current study**
**Normal Cells**	**Bronchial epithelium**	25 - 100	9.3 - 37	5	No effect	[21]
	**Lymphocytes**	**2.7 - 672**	**1 - 250**	**4**	**No effect**	**Current study**
	**Prostrate epithelium (PWR-1E)**	5 - 100	1.8 - 37	40	Not defined	[56]
		10 - 100	3.7 - 37	5	No effect	[19]
	**Epidermal keratinocytes**	5 - 75	1.8 - 28	11 (48 h)	No effect	[14]

The anti-proliferative and apoptotic activity of berberine have been attributed to its concentration-dependent selective accumulation in mitochondria at lower concentration and nuclear as well as cytoplasmic accumulation at higher mitochondria-saturating doses that could interfere with DNA synthesis, perturb cell cycle and sufficient to trigger apoptosis. Our long term cultures of berberine-treated cells also demonstrated a suppressed growth at low doses without any prominent cell death component. Since berberine is a substrate of ATP-driven drug efflux pump [51], it is likely that at saturating concentrations berberine reduces the energy levels of the mitochondria below critical levels resulting in triggering of programmed cell death. This assumption gets strength from the experiments showing berberine effectively synergizes with drug efflux pump inhibitors [52]. Some investigators propose berberine's DNA-binding activity[53] could be responsible for rapid inhibition of DNA synthesis of berberine-treated cells and cell cycle arrest in S phase and G2/M phase [20]. Apart from these direct actions of berberine its inhibitory action on viral oncoproteins (E6/E7) expression via inhibition of AP-1 could be primarily responsible for growth suppression and induction of apoptosis in HPV positive cervical cancer cells. Our observations together with confirmatory annexin V analysis, suggest berberine could antagonize multiple survival and growth promoting mechanisms operating in cervical cancer cells and can induce apoptosis in a dose-dependent manner.

The key biochemical event involved in the induction of apoptosis is activation of caspase3 which is mediated through proteolytic cleavage of procaspase3 via upstream caspases (caspase7/9 or caspase 8). Berberine-treated cells demonstrated activation of caspase3 which also corroborated with proteolytic cleavage of its substrate PARP-1 as early as 6 hours. These apoptotic events were found associated with loss of mitochondrial membrane potential which is the primary mechanism of action of many chemotherapeutic/chemopreventive agents as well as other external apoptotic stimuli [54]. This event is sufficient to release cytochrome C from mitochondrial membrane and execute proteolytic activation of caspases. Nonetheless, direct mitochondrial tropism of berberine through induction of GADD153 levels [26] could also have directly contributed to the loss of mitochondrial potential. Though there could be multiple direct or indirect mechanisms, these observations collectively indicate a potential role of mitochondria in berberine-induced apoptosis.

## Conclusions

In view of potential anti-HPV activity displayed by berberine through inhibition of constitutively active AP-1 as well as its selective, anti-proliferation and cytotoxic effects coupled with pharmacological safety in human, berberine appears to be a promising therapeutic agent for the treatment of cervical cancers.

## Abbreviations

HPV: Human Papillomavirus; AP-1: Activator protein-1; EMSA: electrophoretic mobility shift assay; PARP-1: Poly (ADP-ribose) - polymerase-1; MTT: 3-(4, 5-dimethylthiazol-2-yl)-2; 5-diphenyltetrazolium bromide; FACS: fluorescence-activated cell sorting.

## Competing interests

The authors declare that they have no competing interests.

## Authors' contributions

SM designed, performed experiments, analyzed data, and prepared the manuscript; ACB performed the experimental design, analyzed the data, drafted and wrote the manuscript; SS analyzed the data and helped in manuscript preparation; AT performed experiments of Flow cytometry; SAH reviewed the manuscript; and BCD initiated and planned the project, directed the whole study and revised the manuscript critically. All the authors read and proved the final manuscript.

## References

[B1] Parkin DM The global health burden of infection-associated cancers in the year 2002 Int J Cancer 2006 118 3030 3044 10.1002/ijc.21731 16404738

[B2] Durst M Gissmann L Ikenberg H zur Hausen H A papillomavirus DNA from a cervical carcinoma and its prevalence in cancer biopsy samples from different geographic regions Proc Natl Acad Sci USA 1983 80 3812 3815 10.1073/pnas.80.12.3812 6304740 PMC394142

[B3] Scheffner M Werness BA Huibregtse JM Levine AJ Howley PM The E6 oncoprotein encoded by human papillomavirus types 16 and 18 promotes the degradation of p53 Cell 1990 63 1129 1136 10.1016/0092-8674(90)90409-8 2175676

[B4] Butz K Hoppe-Seyler F Transcriptional control of human papillomavirus (HPV) oncogene expression: composition of the HPV type 18 upstream regulatory region J Virol 1993 67 6476 6486 8411351 10.1128/jvi.67.11.6476-6486.1993 PMC238084

[B5] Milde-Langosch K The Fos family of transcription factors and their role in tumourigenesis Eur J Cancer 2005 41 2449 2461 10.1016/j.ejca.2005.08.008 16199154

[B6] Prusty BK Das BC Constitutive activation of transcription factor AP-1 in cervical cancer and suppression of human papillomavirus (HPV) transcription and AP-1 activity in HeLa cells by curcumin Int J Cancer 2005 113 951 960 10.1002/ijc.20668 15514944

[B7] Bharti AC Shukla S Mahata S Hedau S Das BC Anti-human papillomavirus therapeutics: facts & future Indian J Med Res 2009 130 296 310 19901439

[B8] Gibbs PJ Seddon KR The British Library Studies in Conservation Science 1998 Toronto: University of Toronto Press Inc

[B9] Imanshahidi M Hosseinzadeh H Pharmacological and therapeutic effects of Berberis vulgaris and its active constituent, berberine Phytother Res 2008 22 999 1012 10.1002/ptr.2399 18618524

[B10] Satyavathi GV Gupta AK Tandon N Medicinal Plants of India 1987 New Delhi: Indian Council of Medical Research

[B11] Sun Y Xun K Wang Y Chen X A systematic review of the anticancer properties of berberine, a natural product from Chinese herbs Anticancer Drugs 2009 20 757 769 10.1097/CAD.0b013e328330d95b 19704371

[B12] Lin CC Lin SY Chung JG Lin JP Chen GW Kao ST Down-regulation of cyclin B1 and up-regulation of Wee1 by berberine promotes entry of leukemia cells into the G2/M-phase of the cell cycle Anticancer Res 2006 26 1097 1104 16619512

[B13] Letasiova S Jantova S Cipak L Muckova M Berberine-antiproliferative activity in vitro and induction of apoptosis/necrosis of the U937 and B16 cells Cancer Lett 2006 239 254 262 10.1016/j.canlet.2005.08.024 16229943

[B14] Mantena SK Sharma SD Katiyar SK Berberine inhibits growth, induces G1 arrest and apoptosis in human epidermoid carcinoma A431 cells by regulating Cdki-Cdk-cyclin cascade, disruption of mitochondrial membrane potential and cleavage of caspase 3 and PARP Carcinogenesis 2006 27 2018 2027 10.1093/carcin/bgl043 16621886

[B15] Hwang JM Kuo HC Tseng TH Liu JY Chu CY Berberine induces apoptosis through a mitochondria/caspases pathway in human hepatoma cells Arch Toxicol 2006 80 62 73 10.1007/s00204-005-0014-8 16189662

[B16] Kuo CL Chi CW Liu TY The anti-inflammatory potential of berberine in vitro and in vivo Cancer Lett 2004 203 127 137 10.1016/j.canlet.2003.09.002 14732220

[B17] Eom KS Hong JM Youn MJ So HS Park R Kim JM Kim TY Berberine induces G1 arrest and apoptosis in human glioblastoma T98G cells through mitochondrial/caspases pathway Biol Pharm Bull 2008 31 558 562 10.1248/bpb.31.558 18379040

[B18] Peng PL Hsieh YS Wang CJ Hsu JL Chou FP Inhibitory effect of berberine on the invasion of human lung cancer cells via decreased productions of urokinase-plasminogen activator and matrix metalloproteinase-2 Toxicol Appl Pharmacol 2006 214 8 15 10.1016/j.taap.2005.11.010 16387334

[B19] Mantena SK Sharma SD Katiyar SK Berberine, a natural product, induces G1-phase cell cycle arrest and caspase-3-dependent apoptosis in human prostate carcinoma cells Mol Cancer Ther 2006 5 296 308 10.1158/1535-7163.MCT-05-0448 16505103

[B20] Lin JP Yang JS Lee JH Hsieh WT Chung JG Berberine induces cell cycle arrest and apoptosis in human gastric carcinoma SNU-5 cell line World J Gastroenterol 2006 12 21 28 16440412 10.3748/wjg.v12.i1.21 PMC4077487

[B21] Katiyar SK Meeran SM Katiyar N Akhtar S p53 cooperates berberine-induced growth inhibition and apoptosis of non-small cell human lung cancer cells in vitro and tumor xenograft growth in vivo Mol Carcinog 2008 48 24 37 10.1002/mc.20453 18459128

[B22] Anis KV Rajeshkumar NV Kuttan R Inhibition of chemical carcinogenesis by berberine in rats and mice J Pharm Pharmacol 2001 53 763 768 10.1211/0022357011775901 11370717

[B23] Nishino H Kitagawa K Fujiki H Iwashima A Berberine sulfate inhibits tumor-promoting activity of teleocidin in two-stage carcinogenesis on mouse skin Oncology 1986 43 131 134 10.1159/000226349 3081844

[B24] Kim S Choi JH Kim JB Nam SJ Yang JH Kim JH Lee JE Berberine suppresses TNF-alpha-induced MMP-9 and cell invasion through inhibition of AP-1 activity in MDA-MB-231 human breast cancer cells Molecules 2008 13 2975 2985 10.3390/molecules13122975 19052522 PMC6244848

[B25] Yount G Qian Y Moore D Basila D West J Aldape K Arvold N Shalev N Haas-Kogan D Berberine sensitizes human glioma cells, but not normal glial cells, to ionizing radiation in vitro J Exp Ther Oncol 2004 4 137 143 15500008

[B26] Lin JP Yang JS Chang NW Chiu TH Su CC Lu KW Ho YT Yeh CC Mei D Lin HJ Chung JG GADD153 mediates berberine-induced apoptosis in human cervical cancer Ca ski cells Anticancer Res 2007 27 3379 3386 17970084

[B27] Bharti AC Panigrahi A Sharma PK Gupta N Kumar R Shukla S Bhowmik DM Agarwal SK Guleria S Mehra NK Clinical relevance of curcumin-induced immunosuppression in living-related donor renal transplant: an in vitro analysis Exp Clin Transplant 2010 8 161 171 20565374

[B28] Rosl F Das BC Lengert M Geletneky K zur Hausen H Antioxidant-induced changes of the AP-1 transcription complex are paralleled by a selective suppression of human papillomavirus transcription J Virol 1997 71 362 370 8985358 10.1128/jvi.71.1.362-370.1997 PMC191059

[B29] Lee W Mitchell P Tjian R Purified transcription factor AP-1 interacts with TPA-inducible enhancer elements Cell 1987 49 741 752 10.1016/0092-8674(87)90612-X 3034433

[B30] Scheidereit C Cromlish JA Gerster T Kawakami K Balmaceda CG Currie RA Roeder RG A human lymphoid-specific transcription factor that activates immunoglobulin genes is a homoeobox protein Nature 1988 336 551 557 10.1038/336551a0 2904654

[B31] Kadonaga JT Courey AJ Ladika J Tjian R Distinct regions of Sp1 modulate DNA binding and transcriptional activation Science 1988 242 1566 1570 10.1126/science.3059495 3059495

[B32] Thierry F Spyrou G Yaniv M Howley P Two AP1 sites binding JunB are essential for human papillomavirus type 18 transcription in keratinocytes J Virol 1992 66 3740 3748 1316480 10.1128/jvi.66.6.3740-3748.1992 PMC241159

[B33] Parker JN Zhao W Askins KJ Broker TR Chow LT Mutational analyses of differentiation-dependent human papillomavirus type 18 enhancer elements in epithelial raft cultures of neonatal foreskin keratinocytes Cell Growth Differ 1997 8 751 762 9218869

[B34] Kyo S Klumpp DJ Inoue M Kanaya T Laimins LA Expression of AP1 during cellular differentiation determines human papillomavirus E6/E7 expression in stratified epithelial cells J Gen Virol 1997 78 Pt 2 401 411 9018063 10.1099/0022-1317-78-2-401

[B35] Fukuda K Hibiya Y Mutoh M Koshiji M Akao S Fujiwara H Inhibition of activator protein 1 activity by berberine in human hepatoma cells Planta Med 1999 65 381 383 10.1055/s-2006-960795 10364850

[B36] Luo Y Hao Y Shi TP Deng WW Li N Berberine inhibits cyclin D1 expression via suppressed binding of AP-1 transcription factors to CCND1 AP-1 motif Acta Pharmacol Sin 2008 29 628 633 10.1111/j.1745-7254.2008.00786.x 18430372

[B37] Soto U Das BC Lengert M Finzer P zur Hausen H Rosl F Conversion of HPV 18 positive non-tumorigenic HeLa-fibroblast hybrids to invasive growth involves loss of TNF-alpha mediated repression of viral transcription and modification of the AP-1 transcription complex Oncogene 1999 18 3187 3198 10.1038/sj.onc.1202765 10359524

[B38] Cho BJ Im EK Kwon JH Lee KH Shin HJ Oh J Kang SM Chung JH Jang Y Berberine inhibits the production of lysophosphatidylcholine-induced reactive oxygen species and the ERK1/2 pathway in vascular smooth muscle cells Mol Cells 2005 20 429 434 16404160

[B39] Liang KW Ting CT Yin SC Chen YT Lin SJ Liao JK Hsu SL Berberine suppresses MEK/ERK-dependent Egr-1 signaling pathway and inhibits vascular smooth muscle cell regrowth after in vitro mechanical injury Biochem Pharmacol 2006 71 806 817 10.1016/j.bcp.2005.12.028 16448624 PMC2639653

[B40] Cavigelli M Dolfi F Claret FX Karin M Induction of c-fos expression through JNK-mediated TCF/Elk-1 phosphorylation Embo J 1995 14 5957 5964 8846788 10.1002/j.1460-2075.1995.tb00284.x PMC394715

[B41] Bakiri L Lallemand D Bossy-Wetzel E Yaniv M Cell cycle-dependent variations in c-Jun and JunB phosphorylation: a role in the control of cyclin D1 expression Embo J 2000 19 2056 2068 10.1093/emboj/19.9.2056 10790372 PMC305681

[B42] Passegue E Wagner EF JunB suppresses cell proliferation by transcriptional activation of p16(INK4a) expression Embo J 2000 19 2969 2979 10.1093/emboj/19.12.2969 10856241 PMC203376

[B43] Yang-Yen HF Chambard JC Sun YL Smeal T Schmidt TJ Drouin J Karin M Transcriptional interference between c-Jun and the glucocorticoid receptor: mutual inhibition of DNA binding due to direct protein-protein interaction Cell 1990 62 1205 1215 10.1016/0092-8674(90)90396-V 2169352

[B44] Chong T Chan WK Bernard HU Transcriptional activation of human papillomavirus 16 by nuclear factor I, AP1, steroid receptors and a possibly novel transcription factor, PVF: a model for the composition of genital papillomavirus enhancers Nucleic Acids Res 1990 18 465 470 10.1093/nar/18.3.465 2155400 PMC333449

[B45] Veldman T Horikawa I Barrett JC Schlegel R Transcriptional activation of the telomerase hTERT gene by human papillomavirus type 16 E6 oncoprotein J Virol 2001 75 4467 4472 10.1128/JVI.75.9.4467-4472.2001 11287602 PMC114198

[B46] Kailash U Soundararajan CC Lakshmy R Arora R Vivekanandhan S Das BC Telomerase activity as an adjunct to high-risk human papillomavirus types 16 and 18 and cytology screening in cervical cancer Br J Cancer 2006 95 1250 1257 10.1038/sj.bjc.6603375 17060942 PMC2360573

[B47] Wu HL Hsu CY Liu WH Yung BY Berberine-induced apoptosis of human leukemia HL-60 cells is associated with down-regulation of nucleophosmin/B23 and telomerase activity Int J Cancer 1999 81 923 929 10.1002/(SICI)1097-0215(19990611)81:6<923::AID-IJC14>3.0.CO;2-D 10362140

[B48] Serafim TL Oliveira PJ Sardao VA Perkins E Parke D Holy J Different concentrations of berberine result in distinct cellular localization patterns and cell cycle effects in a melanoma cell line Cancer Chemother Pharmacol 2008 61 1007 1018 10.1007/s00280-007-0558-9 17661039

[B49] Jantova S Cipak L Cernakova M Kost'alova D Effect of berberine on proliferation, cell cycle and apoptosis in HeLa and L1210 cells J Pharm Pharmacol 2003 55 1143 1149 10.1211/002235703322277186 12956905

[B50] Youn MJ So HS Cho HJ Kim HJ Kim Y Lee JH Sohn JS Kim YK Chung SY Park R Berberine, a natural product, combined with cisplatin enhanced apoptosis through a mitochondria/caspase-mediated pathway in HeLa cells Biol Pharm Bull 2008 31 789 795 10.1248/bpb.31.789 18451495

[B51] Shitan N Tanaka M Terai K Ueda K Yazaki K Human MDR1 and MRP1 recognize berberine as their transport substrate Biosci Biotechnol Biochem 2007 71 242 245 10.1271/bbb.60441 17213652

[B52] Stermitz FR Lorenz P Tawara JN Zenewicz LA Lewis K Synergy in a medicinal plant: antimicrobial action of berberine potentiated by 5'-methoxyhydnocarpin, a multidrug pump inhibitor Proc Natl Acad Sci USA 2000 97 1433 1437 10.1073/pnas.030540597 10677479 PMC26451

[B53] Gong GQ Zong ZX Song YM Spectrofluorometric determination of DNA and RNA with berberine Spectrochim Acta A Mol Biomol Spectrosc 1999 55A 1903 1907 10.1016/S1386-1425(99)00053-0 10507886

[B54] Hail N Jr Mitochondria: A novel target for the chemoprevention of cancer Apoptosis 2005 10 687 705 10.1007/s10495-005-0792-8 16133861

[B55] Ho YT Lu CC Yang JS Chiang JH Li TC Ip SW Hsia TC Liao CL Lin JG Wood WG Chung JG Berberine induced apoptosis via promoting the expression of caspase-8, -9 and -3, apoptosis-inducing factor and endonuclease G in SCC-4 human tongue squamous carcinoma cancer cells Anticancer Res 2009 29 4063 4070 19846952

[B56] Choi MS Oh JH Kim SM Jung HY Yoo HS Lee YM Moon DC Han SB Hong JT Berberine inhibits p53-dependent cell growth through induction of apoptosis of prostate cancer cells Int J Oncol 2009 34 1221 1230 10.3892/ijo_00000234 19360335

[B57] Lin CC Yang JS Chen JT Fan S Yu FS Yang JL Lu CC Kao MC Huang AC Lu HF Chung JG Berberine induces apoptosis in human HSC-3 oral cancer cells via simultaneous activation of the death receptor-mediated and mitochondrial pathway Anticancer Res 2007 27 3371 3378 17970083

[B58] Tang F Wang D Duan C Huang D Wu Y Chen Y Wang W Xie C Meng J Wang L Berberine inhibits metastasis of nasopharyngeal carcinoma 5-8F cells by targeting Rho kinase-mediated Ezrin phosphorylation at threonine 567 J Biol Chem 2009 284 27456 27466 10.1074/jbc.M109.033795 19651779 PMC2785675

[B59] Lin CC Kao ST Chen GW Ho HC Chung JG Apoptosis of human leukemia HL-60 cells and murine leukemia WEHI-3 cells induced by berberine through the activation of caspase-3 Anticancer Res 2006 26 227 242 16475703

[B60] Lin HL Liu TY Lui WY Chi CW Up-regulation of multidrug resistance transporter expression by berberine in human and murine hepatoma cells Cancer 1999 85 1937 1942 10223233

[B61] Tan YL Goh D Ong ES Investigation of differentially expressed proteins due to the inhibitory effects of berberine in human liver cancer cell line HepG2 Mol Biosyst 2006 2 250 258 10.1039/b517116d 16880943

[B62] Halimani M Chandran SP Kashyap S Jadhav VM Prasad BL Hotha S Maiti S Dendritic effect of ligand-coated nanoparticles: enhanced apoptotic activity of silica-berberine nanoconjugates Langmuir 2009 25 2339 2347 10.1021/la802761b 19146398

[B63] Lin TH Kuo HC Chou FP Lu FJ Berberine enhances inhibition of glioma tumor cell migration and invasiveness mediated by arsenic trioxide BMC Cancer 2008 8 58 10.1186/1471-2407-8-58 18294404 PMC2275285

[B64] Dvorak Z Vrzal R Maurel P Ulrichova J Differential effects of selected natural compounds with anti-inflammatory activity on the glucocorticoid receptor and NF-kappaB in HeLa cells Chem Biol Interact 2006 159 117 128 10.1016/j.cbi.2005.10.105 16289013

